# Barriers and Facilitators in Secondary Stroke Prevention Among Older Adults: An International Systematic Review of Randomized Controlled Trials

**DOI:** 10.3390/healthcare13243260

**Published:** 2025-12-12

**Authors:** Myrto Pyrrou, Anna Tsiakiri, Konstantinos Vadikolias, Hariklia Proios

**Affiliations:** 1 Department of Neurology, Democritus University of Thrace, 68100 Alexandroupolis, Greece; atsiakir@med.duth.gr (A.T.); kvadikol@med.duth.gr (K.V.); 2 Department of Educational and Social Policy, University of Macedonia, 54636 Thessaloniki, Greece; hproios@uom.edu.gr; 3 Department of Communication Sciences and Disorders, Adelphi University, New York, NY 11530-0701, USA

**Keywords:** secondary stroke prevention, older adults, adherence, barriers and facilitators, education, multidisciplinary care, digital health, rehabilitation, cognitive recovery, healthy aging

## Abstract

**Highlights:**

**What are the main findings?**

- Most studies emphasized that effective secondary stroke prevention in older adults depends on continuous education and empowerment of patients and caregivers, integrated with pharmacological and behavioral management.
- Personalized teaching interventions, digital and home-based monitoring, and multidisciplinary teamwork emerged as key facilitators, whereas poor awareness and limited training opportunities were major barriers.

**What are the implications of the main findings?**

- Educational strategies should be placed at the core of secondary prevention, ensuring that both patients and caregivers understand stroke warning signs, risk factors, and medication adherence.
- Integrating ongoing education with digital follow-up and caregiver involvement can enhance long-term adherence and reduce recurrence risk among older adults.

**Abstract:**

**Background/Objectives:** Secondary stroke prevention is a cornerstone of long-term recovery and healthy aging among older adults, yet adherence to preventive strategies remains suboptimal. This global systematic review aimed to synthesize evidence from randomized controlled trials evaluating interventions that support sustained secondary prevention in older adults after stroke. **Methods:** A systematic search of PubMed and Scopus databases was conducted up to April 2025, following PRISMA 2020 guidelines and registered in PROSPERO (CRD420251177501). Eligible studies included randomized controlled trials targeting adults aged 60 years or older and assessing pharmacological, behavioral, educational, rehabilitative, or technology-assisted interventions for stroke recurrence prevention. Data were narratively synthesized due to study heterogeneity, and methodological quality was appraised using the Cochrane RoB 2 tool. **Results:** Seventeen randomized trials involving approximately 17,000 participants met the inclusion criteria. Multicomponent programs integrating medication management, behavioral education, exercise, cognitive rehabilitation, and digital support consistently improved adherence, vascular risk control, and quality of life. Pharmacological strategies alone showed limited or transient benefits, underscoring the importance of patient education and sustained follow-up. Common barriers included low motivation, cognitive decline, and technological challenges, while key facilitators were personalized education, multidisciplinary coordination, and culturally adapted implementation. **Conclusions:** Effective secondary stroke prevention in older adults depends on integrated, person-centered models that combine education, behavioral reinforcement, and technology-assisted monitoring. Structured, continuous educational programs, embedded within rehabilitation and primary care, emerge as the most promising pathway to improve adherence, reduce recurrence, and promote active, autonomous aging.

## 1. Introduction

Stroke remains one of the leading causes of death and long-term disability worldwide, representing a growing challenge for aging populations and healthcare systems. According to the Global Burden of Disease Study 2021, more than 12 million people experience a stroke each year, with over 6.5 million deaths and millions more living with residual physical and cognitive impairment, profoundly affecting independence and quality of life [1,2]. Epidemiological registry data further show that the annual cumulative incidence of acute cerebrovascular disease can exceed 200 cases per 100,000 population, with reported rates of 218 per 100,000 in men and 127 per 100,000 in women in a large Spanish population-based study [3]. Older adults experience nearly 70% of all stroke events and face disproportionately higher rates of functional dependency, institutionalization, and recurrent stroke. Beyond its clinical impact, stroke places a considerable economic burden on healthcare systems, exceeding €60 billion annually in Europe alone [4,5]. As populations age and life expectancy increases, the prevalence of recurrent stroke is expected to rise, further emphasizing the need for effective long-term secondary prevention strategies.

Secondary prevention plays a critical role in reducing the long-term burden of stroke by preventing recurrent events among individuals who have already experienced a cerebrovascular episode. While primary prevention targets first-ever stroke risk, secondary prevention focuses on minimizing recurrence through optimal management of vascular risk factors and lifestyle modification [6]. Recurrent strokes account for nearly one in four stroke cases worldwide and are typically more disabling, fatal, and costly than initial events, highlighting the importance of effective secondary prevention as a critical public health priority [2,7].

Evidence-based guidelines from the American Heart Association/American Stroke Association [6] and the Stroke Action Plan for Europe 2018–2030 [7] emphasize the need for a multifactorial, integrated approach to secondary prevention. Recommended strategies include optimal blood pressure and lipid control, antithrombotic therapy, atrial fibrillation management, diabetes control, smoking cessation, regular physical activity, and adherence to a heart-healthy diet. However, pharmacological strategies alone are insufficient without behavioral reinforcement, patient education, and support for sustained adherence [8]. Despite strong evidence and clear clinical guidelines, major gaps remain in implementation: adherence to antiplatelet or antihypertensive therapy often declines within months after discharge, while lifestyle changes frequently prove difficult to sustain over time [9,10]. Importantly, little is known about which strategies are most effective for sustaining long-term secondary prevention in older adults.

Effective secondary prevention also requires coordinated collaboration across the healthcare system. Integration of neurologists, primary care physicians, nurses, rehabilitation specialists, and caregivers is essential to maintain continuity of care and consistent monitoring of vascular risk factors [7,11]. Multidisciplinary and person-centered models are particularly important for older adults, in whom multimorbidity, polypharmacy, cognitive impairment, and functional limitations add complexity to post-stroke management. Strengthening long-term support across care settings is therefore critical for improving adherence and sustaining secondary prevention outcomes in aging populations.

Overall, secondary prevention is essential for reducing recurrent stroke, improving long-term functional outcomes, and lowering healthcare costs. Despite strong evidence supporting these strategies, their implementation in real-world practice remains inconsistent, particularly among older adults. Understanding which approaches are most effective and feasible is therefore critical for improving long-term stroke care and supporting sustained secondary prevention.

In this context, older adults represent the population group most affected by stroke, yet they remain underrepresented in clinical research and secondary prevention initiatives. Aging is associated with multimorbidity, frailty, cognitive impairment, and social isolation, which make adherence to pharmacological and lifestyle recommendations particularly challenging [12,13]. Complex medication regimens and polypharmacy further increase the risk of non-adherence and adverse effects, while limited mobility, transportation barriers, and reduced health literacy restrict access to follow-up and rehabilitation services [14,15]. As a result, older adults often receive less intensive risk-factor management and participate less frequently in structured prevention programs compared with younger patients [12,16]. These disparities highlight the need for tailored, age-sensitive strategies that integrate medical, behavioral, and social components to support sustained adherence and improve outcomes in older stroke survivors.

Despite the existence of robust international guidelines, the implementation of secondary prevention in older adults remains limited and inconsistently evaluated. Most randomized controlled trials have focused on younger or mixed-age populations, leaving uncertainty about the most effective strategies for older stroke survivors. This gap underscores the need for a systematic synthesis of available evidence to determine which approaches are most effective for older stroke survivors.

Therefore, this systematic review aims to synthesize evidence from randomized controlled trials evaluating secondary stroke prevention strategies in older adults, including pharmacological, behavioral, educational, rehabilitative, technology-assisted and physical-activity-based approaches. By identifying key facilitators and barriers to sustained engagement and recovery, the review seeks to inform clinical practice, support evidence-based policymaking, and guide the development of more effective, age-sensitive secondary prevention strategies.

## 2. Materials and Methods

### 2.1. Literature Searches

Two investigators (M.P. and A.T.) conducted a literature search of two databases (PUBMED and SCOPUS) to trace all relevant studies up to 7 April 2025. All retrieved articles were also manually searched for further potentially eligible articles. Any disagreements regarding study screening, inclusion, or data extraction were resolved through discussion with the other two investigators (H.P. and K.V.) until a consensus was reached.

#### Search Strategy

A comprehensive literature search was conducted to identify studies exploring barriers and facilitators related to secondary stroke prevention among elderly people or older adults. Although the initial search terms were tailored to identify studies reporting barriers and facilitators, all retrieved records were subsequently screened in full text, and all randomized controlled trials on secondary stroke prevention were assessed for eligibility regardless of whether barrier-related terminology appeared in the title, abstract, or keywords. This approach ensured that no intervention RCTs were missed due to the specificity of the initial search terms. Two major electronic databases, PubMed and Scopus, were systematically searched to ensure a broad coverage of relevant literature.

We restricted electronic searches to PubMed and Scopus because together they provide the broadest international coverage of biomedical and multidisciplinary research relevant to secondary stroke prevention. PubMed offers comprehensive indexing of clinical trials, neurology and geriatric medicine studies, while Scopus captures a wider international range of journals, public health research, rehabilitation science, and digital health interventions not covered in PubMed. To reduce the risk of missing eligible studies, we also conducted manual reference checking of all included articles.

The PubMed search was performed using the following search string:

((barriers) OR (facilitators)) AND ((secondary) AND (stroke prevention)) AND ((elderly) OR (older adults))

This search strategy combined key terms related to perceived barriers and facilitators with terms addressing secondary stroke prevention in elderly populations. No date restrictions were applied. Only studies published in English were included.

In Scopus, the following query was used to retrieve peer-reviewed journal articles:

((ALL(“barriers”) OR ALL (“facilitators”) AND ALL (“secondary stroke prevention”) AND ALL (elderly) OR ALL (“older adults”)) AND (LIMIT-TO (LANGUAGE,”English”)) AND (LIMIT-TO (PUBSTAGE,”final”)) AND (LIMIT-TO (DOCTYPE,”ar”)))

The Scopus search was limited to final-stage, English-language research articles to ensure the inclusion of high-quality, peer-reviewed evidence.

In addition, manual reference checking was performed to minimize the risk of missing relevant RCTs not captured by the database search terms.

The review protocol was registered in the PROSPERO International Prospective Register of Systematic Reviews (registration number CRD420251177501), available at https://www.crd.york.ac.uk/PROSPERO/view/CRD420251177501 (accessed on 7 December 2025).

### 2.2. Selection Criteria

We included randomized controlled trials (RCTs) focusing on secondary prevention after ischemic or hemorrhagic stroke or transient ischemic attack (TIA) in adults aged 60 years and above. Eligible studies assessed interventions targeting medication adherence, lifestyle modification, education, rehabilitation, or digital support tools. Both full and pilot/feasibility RCTs were retained when they targeted stroke recurrence prevention in older adults and met minimum methodological quality standards. Pilot or feasibility studies that lacked randomization or had insufficient methodological detail were excluded.

This systematic review was structured according to the PICO (Population, Intervention, Comparator, Outcome) framework (Table 1) to ensure a clear and transparent formulation of the research question and eligibility criteria.

This PICO framework guided both the literature search and the study selection process, ensuring consistency and methodological rigor throughout the review.

**Figure 1:**
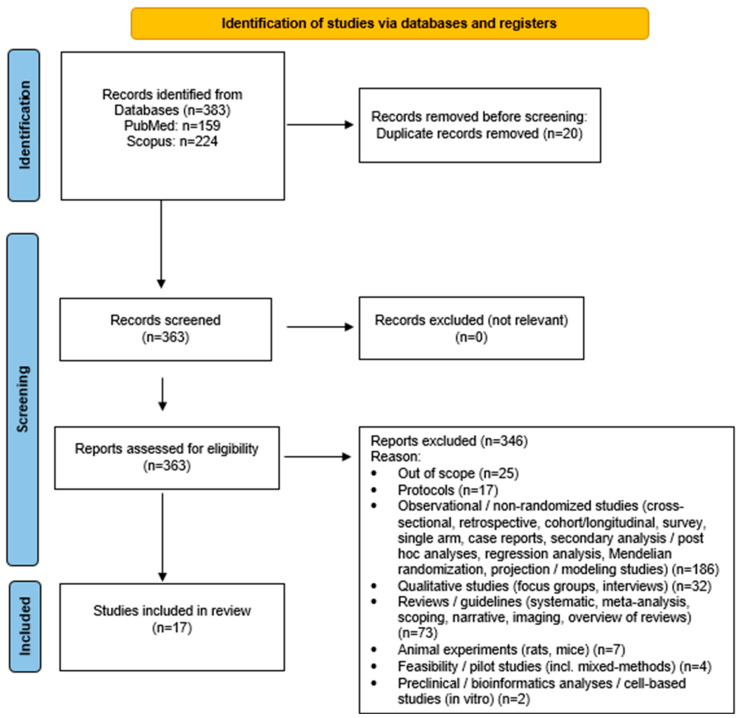
Study flow diagram (PRISMA flowchart).

Only full-text articles written in English were considered. All potentially relevant records were screened in full text because the abstracts frequently lacked sufficient methodological detail to determine eligibility. All 383 retrieved records were directly assessed in full text because the abstracts did not provide sufficient methodological detail to reliably determine eligibility. This full-text approach was applied to ensure that no potentially relevant RCTs were incorrectly excluded based on incomplete abstract information. All identified articles were independently assessed in full text by two reviewers. Any disagreements were resolved through discussion with a third reviewer, and a fourth senior reviewer oversaw the process to ensure methodological consistency across all stages. The screening and selection process is summarized in Figure 1 (PRISMA flow diagram) [17], which provides an overview of the number of records identified, screened, excluded, and included in the final synthesis. To enhance transparency, exclusion reasons were grouped into broader methodological categories (e.g., observational designs, qualitative studies, feasibility studies). All counts in the PRISMA diagram correspond to the final full-text assessments, and categories were aggregated to improve clarity and avoid excessive fragmentation. Studies were excluded if they were non-randomized, observational, qualitative, preclinical, or animal experiments, as well as reviews, meta-analyses, and protocols. Although barriers and facilitators are traditionally examined through qualitative or mixed-methods research, RCTs frequently report implementation-related elements such as adherence challenges, feasibility issues, contextual factors, and participant engagement. In this review, such information was extracted strictly as reported by the authors of each RCT, without inferring additional concepts beyond the original study data.

### 2.3. Data Extraction

Data extraction was performed using a predefined Excel sheet specifically designed for this review. For each included study, we recorded the author, year of publication, country, study design, population characteristics, intervention type, comparator, duration, outcomes assessed, and main findings. A detailed summary of all included studies is provided in the results section. This structured approach enabled a consistent and systematic comparison across all 17 randomized controlled trials included in the review, ensuring accuracy during data synthesis. Both full and pilot/feasibility RCTs were included, provided that randomization procedures were clearly described.

### 2.4. Data Analysis

No statistical analysis or meta-analysis was performed due to the high heterogeneity observed among studies. A descriptive synthesis was therefore conducted to summarize key study characteristics and outcomes. The included randomized controlled trials were grouped thematically according to the main focus of the intervention, such as medication adherence, education, rehabilitation, physical activity, psychological support, and technology-based approaches. Pilot and feasibility RCTs were analyzed narratively to avoid over-interpretation of preliminary findings, while fully powered RCTs were used to identify robust trends and common patterns across interventions.

### 2.5. Risk of Bias Assessment

The quality of the included randomized controlled trials was assessed using the revised Cochrane Risk of Bias 2 (RoB 2) tool [18], following the guidance of the Cochrane Handbook for Systematic Reviews of Interventions [19]. This tool evaluates five domains: randomization process, deviations from intended interventions, missing outcome data, measurement of the outcome, and selection of the reported result.

## 3. Results

### 3.1. Database Searches and Quality Assessment of Included Studies

A total of 383 records were initially retrieved from the database searches: 224 from Scopus and 159 from PubMed. All 383 retrieved records were screened in full text to determine eligibility, as the abstracts alone were not sufficient to assess the inclusion criteria. After removing 20 duplicate entries, 363 records were evaluated based on the predefined inclusion and exclusion criteria. Among these, 346 studies were excluded because they did not meet the eligibility requirements, most commonly due to non-randomized design, ineligible population age, unrelated intervention focus, or insufficient outcome data. Additional exclusion reasons are summarized in Figure 1 (PRISMA flow diagram) [17]. Consequently, a total of 17 articles met the inclusion criteria and were incorporated into the final synthesis (Figure 1). The overall judgment for each study is illustrated in Table 2, with detailed domain-level judgments provided in Supplementary Table S2. Most trials were rated as having some concerns of bias, primarily due to limited information regarding allocation concealment and blinding procedures. No study was classified as having a high risk of bias.

### 3.2. Study Origin

The included studies originated from a wide range of geographical regions. The majority of the studies were conducted in Europe, accounting for five studies from Sweden, Germany (in collaboration with the UK), the Netherlands, Ireland, and Denmark [20,21,22,23,24]. Asia was represented with four studies from Japan, China, India, and Malaysia [25,26,27,28]. The United States contributed five studies [29,30,31,32,33], while North America (Canada) was represented in two studies [34,35]. Additionally, one study originated from Africa (Ghana) [36]. This distribution reflects contributions from multiple continents, with studies conducted across Europe, Asia, North America and Africa.

**Table 2:** Quality of included randomized trials assessed via Risk of Bias (RoB 2).

Included Randomized Trials	Domain 1— Randomization	Domain 2— Deviations	Domain 3— Missing Data	Domain 4— Measurement	Domain 5—Selection	Overall
Appalasamy 2020 [25]	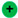	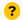	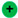	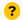	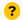	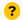
Babu 2024 [26]	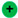	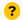	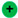	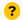	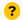	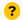
Damush 2011 [29]	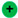	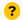	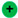	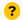	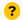	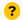
Deijle 2024 [20]	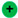	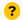	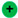	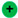	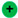	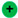
Doogue 2023 [21]	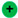	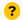	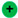	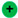	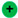	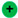
Feldman 2020 [30]	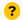	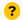	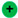	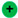	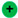	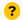
Geary 2019 [22]	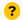	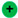	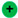	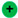	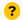	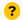
Hornnes 2011 [23]	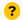	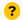	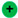	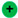	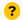	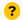
MacKay-Lyons 2022 [34]	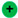	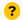	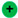	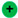	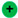	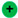
Perera 2025 [35]	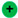	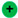	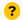	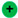	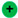	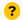
Sajatovic 2018 [31]	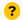	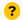	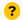	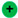	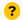	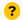
Sarfo 2023 [36]	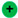	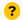	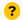	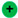	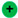	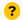
Skidmore 2015 [32]	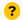	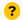	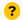	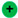	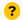	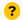
Studer 2021 [24]	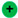	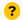	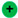	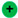	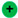	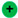
Towfighi 2020 [33]	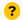	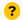	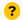	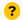	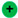	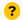
Yamagami 2024 [27]	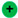	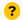	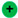	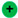	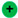	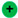
Yan 2021 [28]	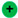	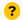	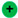	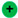	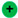	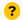

Notes: 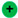 = Low risk of bias/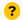 = Some concerns.

### 3.3. Study Design

All included studies employed a randomized controlled design, ensuring a high level of methodological rigor across the review. Among the seventeen studies, the majority were standard randomized controlled trials (RCTs) with variations such as open-label, single-blind, assessor-blinded, or multicenter designs. Several studies utilized parallel two-group structures or three-arm comparisons, while others adopted pragmatic or community-based cluster-randomized approaches to reflect real-world implementation contexts. Also, two studies were categorized as pilot or feasibility RCTs; both implemented randomization procedures and met predefined methodological quality criteria. One primarily evaluated intervention feasibility and acceptability, while the other applied a mixed-methods feasibility framework as part of a randomized design to inform a subsequent large-scale trial. The duration of follow-up across studies ranged from short-term pilot evaluations (3–6 months) to longitudinal interventions extending up to one year.

### 3.4. Population

Altogether, the studies represented a population of roughly 17,000 individuals, most of whom were stroke and transient ischemic attack (TIA) survivors. Among these, one study involved healthcare professionals as participants, whose reported outcomes were derived from 12,766 patients managed in primary care settings [22]. The participants covered a wide age range, from early adulthood (around 20 years) to 80 years, with most samples clustered between 60 and 70 years, reflecting an older population at elevated vascular risk. Sex-specific data were reported in most trials, showing an overall balanced gender distribution, with women representing approximately half of the total sample.

In terms of stroke type, the majority of studies included participants with ischemic stroke or TIA, while a smaller number incorporated mixed cohorts of ischemic and hemorrhagic stroke survivors. Only a few trials provided detailed stratification by stroke subtype. The rehabilitation phase at enrollment varied, although most participants were in the chronic or subacute stages of recovery. Several studies included patients within the first 2–3 months post-stroke, whereas others recruited long-term survivors several years after the index event, highlighting the wide variation in post-stroke recovery stages represented across the studies (Table 3).

### 3.5. Care Setting

The care settings across studies ranged widely, including primary care practices, tertiary hospitals, community clinics, rehabilitation facilities, and home-based programs. Interventions were implemented in diverse healthcare contexts, from acute inpatient rehabilitation to community-based self-management and home follow-up.

Notably, many of the included trials specifically targeted underserved or socioeconomically diverse populations, including stroke survivors living in rural areas of Canada [34], minority groups such as African American and Hispanic patients in the United States [30], and low-education communities in Asia [28]. These groups often had limited access to healthcare and follow-up services.

### 3.6. Data Collection

Data collection methods differed across the included studies, combining clinical, laboratory, imaging, and self-reported measures to ensure comprehensive outcome assessment. Most studies used standardized clinical and laboratory evaluations, including measurements of blood pressure, lipid levels, glycemic indices, and anthropometric parameters such as body mass index and waist circumference. In addition, several trials used neuroimaging methods, such as MRI, CT angiography, carotid ultrasound, and digital subtraction angiography, to evaluate cerebrovascular status and track potential disease progression.

In parallel, structured questionnaires and validated scales were widely applied to evaluate medication adherence, quality of life, physical activity, cognitive function, and mood. The most frequently used tools included the Morisky and Hill–Bone adherence scales, EQ-5D, MoCA, HADS, Hamilton Depression Scale [36], and the Medication Understanding and Use Self-Efficacy (MUSE) scale [25]. In addition, self-report surveys, home blood pressure logs, and digital monitoring tools (e.g., the SINEMA and BP:Together mobile platforms) were also used to support longitudinal follow-up and remote data collection [21,28].

Several studies also incorporated qualitative or mixed-methods approaches, such as semi-structured interviews, focus groups, and patient feedback questionnaires, which were often used to explore behavioral barriers and assess intervention feasibility. Follow-up assessments were typically conducted at baseline, 3, 6, and 12 months, with some trials extending up to one year post-enrollment.

### 3.7. Intervention/Focus

The interventions addressed several aspects of secondary prevention, mostly focusing on medication adherence, vascular risk factor control, and lifestyle modification. A number of trials examined pharmacological optimization strategies, such as the use of fixed-dose combination therapy (polypill) [36], the addition of cilostazol or low-dose rivaroxaban to standard regimens [35], and case management or audit-and-feedback approaches to improve prescription practices in primary care [22]. Others focused on behavioral and educational strategies, including the HEALS [33] and TEAM self-management programs [31], which combined patient education, goal setting, and peer support to encourage long-term adherence and risk reduction.

Technology-assisted and mobile health (mHealth) interventions were also frequently represented. Examples include the SINEMA [28] and BP: Together platforms [21], which provided automated reminders, remote monitoring, and tailored health messages delivered via smartphone or voice calls. Several studies also introduced nurse- [23,30] or physician-led [22,24,26,29] transitional care programs, involving home visits, telephone follow-ups, and personalized lifestyle counseling. In addition, structured exercise and rehabilitation-based interventions, ranging from aerobic and strength training [20,29,34] to gamified cognitive exercises [24], were incorporated to enhance both physical recovery and cardiovascular health.

### 3.8. Barriers

#### 3.8.1. Methodological Barriers

A number of methodological and implementation barriers were reported across the included studies. The most common challenges involved limited sample sizes, short intervention durations, and single-center designs, which restricted the generalizability of findings. Two of the included trials were pilot or feasibility randomized controlled studies, both underpowered to detect meaningful clinical effects or sustained behavioral change. High baseline control of vascular risk factors in some populations also created a ceiling effect, reducing the potential for further improvement.

#### 3.8.2. Real-World Barriers

Adherence and fidelity issues were frequently noted. In several studies, follow-up intensity was lower than planned, phone call adherence rates were below 60%, and medication or exercise adherence declined over time. In interventions involving healthcare professionals, limited reinforcement, lack of continuity, and one-off feedback were cited as obstacles to sustained practice change. Cultural and linguistic diversity also posed challenges, particularly in multicultural or low-literacy populations.

Technological and logistical constraints were reported in community- and mHealth-based trials, including inconsistent mobile phone access, variable internet connectivity, and user fatigue from frequent digital prompts. Some participants, particularly older adults, experienced challenges in navigating digital tools, while COVID-19-related disruptions affected recruitment, data collection, and intervention delivery in multiple studies [21,35,36].

Several pharmacological and behavioral interventions faced treatment-specific limitations. For example, some trials reported higher adverse event rates or intolerance to agents such as cilostazol [27], suboptimal medication intensity (e.g., low-dose statins) [28], and limited physician adjustment of therapy when blood pressure goals were unmet. Behavioral programs were sometimes too brief (≤6 weeks), lacked family or community engagement, or provided insufficient incentives to maintain participation.

Finally, self-report bias and incomplete blinding were recurrent methodological issues, particularly in lifestyle and home-based interventions. Attrition rates were higher than expected in some studies, and fidelity variability among facilitators occasionally weakened intervention delivery. Overall, these barriers underscore the practical and operational difficulties of sustaining long-term, community-based secondary prevention strategies among diverse stroke populations.

### 3.9. Facilitators

Several facilitators supported the successful implementation and feasibility of the included interventions. Strong study designs, such as multicenter coordination, blinded endpoint assessment, and integration with existing healthcare systems, were common features that enhanced methodological quality. High adherence and retention were frequently observed, with completion rates exceeding 80–90% in multiple trials, and follow-up fidelity also remaining high. Simplified medication regimens, such as once-daily fixed-dose combinations, and low-cost, mHealth-based delivery models contributed to sustained participation, even in low-resource or pandemic-affected settings [36].

Provider engagement and structured implementation strategies were additional strengths. Several interventions incorporated provider training, audit-and-feedback systems, or incentive mechanisms that promoted continuity of care and reinforced evidence-based prescribing practices [22]. Nurse- and physician-led programs achieved high fidelity, often above 85–95% of planned visits, and demonstrated the feasibility of embedding secondary prevention initiatives into routine primary-care workflows.

Adapting interventions to local culture and context was another recurring facilitator. Programs that delivered education or counseling in local languages [33], used aphasia-friendly materials [21], or employed bilingual staff (English/Spanish) [30] achieved better patient engagement and improved accessibility across diverse populations. Group-based and peer-supported components, such as the HEALS [33] and TEAM programs [31], encouraged interaction, goal setting, and mutual motivation, further improving adherence and satisfaction.

Digital and technology-assisted interventions were also well accepted. Mobile and text-based platforms like SINEMA [28] and BP:Together [21] facilitated remote follow-up, automated reminders, and personalized feedback. Users generally reported that the platforms were convenient and easy to use.

Finally, programs with clear theoretical grounding and structured design tended to show better fidelity and patient outcomes. Behavioral models based on social cognitive or self-efficacy frameworks, clear treatment protocols, and attention-control comparators contributed to consistent delivery and reliable evaluation.

## 4. Discussion

### 4.1. Overview of Main Findings

The findings of this systematic review reveal a diverse yet converging base of evidence on secondary stroke prevention among older adults, highlighting both the progress and the ongoing challenges in translating evidence-based interventions into sustained long-term clinical benefit. Across the 17 included randomized controlled trials, most interventions targeted vascular risk factor control [6], particularly blood pressure, lipid levels, and medication adherence, as well as lifestyle modification and self-management behaviors. Despite methodological heterogeneity, the majority of studies showed that secondary prevention can be feasibly implemented across a diverse range of care settings, including primary care, community programs, and home-based or technology-supported interventions.

A recurring observation was that improvements were most pronounced when interventions were multicomponent, well-structured, and integrated into routine care. Programs combining medication management with lifestyle counseling or mHealth support (e.g., mobile apps, text reminders, or remote monitoring) often led to better adherence, lower systolic and diastolic blood pressure, improved quality of life, and, in some cases, reduced stroke recurrence and mortality. These outcomes were evident even in low-resource or rural settings, suggesting that technology-assisted or nurse-led approaches may be scalable and potentially cost-effective when embedded within existing healthcare frameworks.

Conversely, single-focus interventions, such as audit-and-feedback mechanisms [22] or fixed-dose combination therapies (polypill) [36], often yielded limited or short-lived effects. The audit-based intervention directed at primary care physicians, for example, did not improve medication dispensing rates 18 months later, and polypill therapy failed to show superiority over standard care in controlling lipid levels or blood pressure, with a higher adverse-event burden. Similarly, pharmacological trials testing agents such as cilostazol [27] or low-dose rivaroxaban [35] showed acceptable safety and feasibility but were underpowered to demonstrate definitive efficacy. Overall, these findings suggest that pharmacological optimization alone is insufficient without behavioral reinforcement and ongoing monitoring. Such follow-up should be shared across disciplines, with physicians overseeing medical management, educators and rehabilitation specialists delivering structured, person-centered education, and stroke support organizations providing community-based continuity and motivation for long-term adherence, through ongoing interdisciplinary communication and feedback loops.

Behavioral and self-management interventions demonstrated promising outcomes in terms of adherence, self-efficacy, and psychological well-being. Structured programs that incorporated goal setting, feedback, and social or family support, as seen in trials such as TEAM and SINEMA [28,31], produced measurable gains in medication adherence, diet, physical activity, and health-related quality of life. These approaches may be particularly beneficial for marginalized or socially isolated stroke survivors who face limited access to healthcare services and require proactive, community-based strategies to sustain secondary prevention efforts.

Interventions using gamification, precommitment strategies, or video narratives further enhanced engagement and motivation, particularly among community-dwelling survivors. Taken together, these findings indicate consistent patterns across trials and underscore the importance of tailoring programs to individual and contextual needs, reinforcing patient agency in long-term recovery and risk reduction.

Exercise and rehabilitation-based interventions, including aerobic, strength, and cognitive training, were associated with meaningful improvements, particularly in executive function and apathy reduction, although effects on physical performance metrics such as BMI or VO_2_peak were inconsistent. Importantly, the feasibility and acceptability of such programs were generally reported as high, even among older adults, supporting their inclusion in comprehensive secondary prevention models.

Taken together, the evidence indicates that effective secondary stroke prevention in older adults relies on multilevel, patient-centered, and interdisciplinary approaches. Interventions that blend pharmacological optimization with behavioral reinforcement, digital support, and culturally sensitive education appear most capable of sustaining risk reduction and improving long-term outcomes. However, despite encouraging findings, variability in intervention duration, follow-up intensity, and methodological rigor limits the comparability of results. These limitations in reporting and intervention heterogeneity point to the need for standardized frameworks and core outcome sets to ensure durable impact across diverse healthcare contexts.

### 4.2. Comparison with Existing Literature

#### 4.2.1. Guideline Alignment

International and regional stroke prevention guidelines consistently emphasize the integration of medical, behavioral, and systems-level strategies to reduce recurrent events, an approach that closely mirrors the findings of this review. The American Heart Association/American Stroke Association (AHA/ASA 2021) guidelines identify optimal blood pressure and lipid control, antithrombotic therapy, and lifestyle modification as the core pillars of secondary prevention [6]. Similarly, the Stroke Action Plan for Europe 2018–2030 [7] underscores the need for coordinated, multidisciplinary care models that extend beyond the acute phase and into long-term community follow-up. Consistent with these recommendations, the present synthesis reinforces these priorities, showing that multicomponent interventions that combine pharmacological optimization with behavioral reinforcement and patient education are associated with consistent benefits in adherence, quality of life, and risk reduction. Moreover, growing evidence for feasibility and potential scalability in rural and low-resource settings supports the WHO’s global call for equitable access to post-stroke preventive care [11,37].

From a public health and policy perspective, the current findings align with the broader European framework promoted by the Stroke Alliance for Europe (SAFE) and the World Health Organization in recognizing secondary prevention as both a clinical and an economic imperative. The SAFE report “At What Cost” [5] and subsequent analyses by Luengo-Fernández et al. [4] estimated that stroke imposes more than €60 billion annually in combined healthcare and productivity losses across Europe, a burden projected to increase substantially without stronger prevention and rehabilitation infrastructure. In this context, the evidence for nurse-led, digital, and community-based approaches in older adults is particularly relevant, as these models can bridge the gap between guideline recommendations and real-world implementation. Aligning local initiatives with these international priorities by embedding patient-centered, technology-enabled, and interdisciplinary care into national stroke strategies is central to achieving durable, equitable outcomes in secondary stroke prevention.

#### 4.2.2. Adherence and Self-Management in Older Adults

Medication adherence and sustained self-management remain central challenges in secondary stroke prevention among older adults. Despite clear evidence that optimal control of vascular risk factors reduces recurrent events, adherence to prescribed medications and lifestyle recommendations often declines over time, particularly among individuals with multimorbidity, polypharmacy, or cognitive limitations [10,11,38]. Psychological and behavioral barriers, including low self-efficacy, apathy, or concerns about adverse effects, further compromise adherence, while limited social support and caregiver involvement exacerbate this decline [9,39]. Previous research consistently shows that education alone is insufficient and that sustainable adherence requires structured behavioral and motivational components, a conclusion that remains valid across conditions [40,41].

At the same time, evidence from recent trials suggests that adherence improves when education is continuous, practical, and connected to patients’ daily lives. Structured programs that combine goal setting, feedback, and family participation help sustain behavioral change and motivation beyond the clinical setting [10,39]. Nurse-led and community-based interventions, such as SINEMA and TEAM, show that personalized coaching and peer support can maintain blood pressure control and medication adherence even in resource-limited environments [28,31]. Together, these findings highlight that adherence is best maintained when educational support is continuous and embedded within meaningful human relationships. Moreover, cultural and linguistic diversity emerged as a recurring barrier to adherence, particularly among low-literacy or multilingual populations. Future interventions should therefore prioritize culturally adapted education materials and bilingual delivery models to ensure accessibility and engagement across diverse patient groups [28,30].

#### 4.2.3. Digital and Technology-Assisted Care

Digital innovation has emerged as a critical complement to traditional models of secondary stroke prevention, especially for older adults who face barriers related to geography, mobility, or healthcare access [42]. mHealth tools, telemonitoring, and remote coaching programs have been shown to extend the reach of follow-up care, improve medication adherence, blood pressure control, and engagement in lifestyle modification [43,44]. At the same time, more recent evidence highlights the potential of digital supports, such as mHealth reminders and telemonitoring, while also documenting access and usability barriers that can blunt their impact, particularly in stroke populations [45,46,47]. Beyond convenience, digital systems foster continuous communication between patients and clinicians, allowing early detection of blood pressure fluctuations, medication lapses, or mood changes that may otherwise go unnoticed [48]. However, across the included trials and supporting literature, technology alone was insufficient; sustained engagement, digital literacy, and patient–clinician trust remained decisive factors for long-term use and benefit [49,50].

Recent trials integrating telehealth into nurse-led and primary care networks, such as the SINEMA and BP@Home models, highlight how digital tools can empower both patients and healthcare teams [28,51]. In these systems, structured communication, automated reminders, cloud-based dashboards, and personalized feedback loops help maintain motivation and accountability while reducing the burden of travel and clinical workload. Importantly, pragmatic care models embedded in primary care and nurse-led programs that combine digital support with personalized guidance, education and community-based follow-up demonstrate promising scalability and cost-effectiveness, particularly in rural or resource-limited regions where in-person visits are difficult to sustain [28,52]. However, broadband access and device usability barriers, such as low digital literacy, sensory impairments, and reduced confidence with technology, can limit their effectiveness and inclusion for the oldest or most vulnerable patients, underscoring that digital health must be designed for, not merely delivered to, older users [49,50].

Looking ahead, technology-assisted care will play an increasingly central role in long-term stroke prevention. However, the evidence suggests that digital tools are most effective when they complement, rather than replace, human interaction, helping older adults bridge gaps in access and confidence. To achieve equitable and lasting impact, digital interventions should be integrated into interdisciplinary, literacy-sensitive, and relationship-centered care models that combine the precision of data-driven monitoring with the empathy and adaptability of human support. Programs that blend digital follow-up with nurse outreach, caregiver participation, and ongoing feedback appear best positioned to sustain adherence, enhance quality of life, and ensure that older adults fully benefit from the potential of digital health innovations.

#### 4.2.4. Psychosocial and Cognitive Factors

In older stroke survivors, cognitive and psychosocial changes often determine whether risk-reduction plans become daily habits. Recent guidance reports that post-stroke cognitive impairment (PSCI) is common, affecting approximately 40–50% of survivors within the first year, and can weaken executive control, attention, memory, and planning, which are the very skills needed for medication management, blood pressure self-monitoring, and lifestyle change [53]. These deficits frequently coexist with depression, fatigue, anxiety, and apathy, each contributing to eroding self-efficacy and adherence over time. In our synthesis, programs that paired risk-factor management with targeted cognitive-behavioral support achieved more durable engagement than information-only approaches [54].

Cognitive rehabilitation after stroke has shown small but meaningful benefits in selected domains, mainly in memory and strategy use, though results often fade with time and rarely generalize to formal tests. This highlights the need for clearer outcome sets and stronger research designs [54,55,56]. By contrast, strategy-based, metacognitive approaches, such as goal management training, external memory aids, and structured problem-solving, are more likely to translate into everyday function when they are embedded in real-world tasks (e.g., medication scheduling, blood pressure logging, appointments) and delivered by interdisciplinary teams. In this context, such approaches become far more relevant and sustainable for older stroke survivors [55,57]. However, in real-world settings, access to interdisciplinary follow-up after discharge remains limited for many stroke survivors, as care is often confined to a neurologist or primary care physician. This gap underscores the importance of developing scalable community or telehealth-based models that can extend the benefits of multidisciplinary support beyond the hospital setting.

Addressing post-stroke apathy, a frequent yet modifiable barrier to participation, within such interventions may further enhance motivation and adherence. In one randomized inpatient study, adding strategy training to usual rehabilitation maintained lower apathy levels over 3–6 months, supporting the routine incorporation of motivational and planning strategies into secondary prevention pathways [32]. Early remote-delivery pilots combining computerized cognitive exercises with metacognitive coaching show good feasibility for extending these supports beyond the clinic, offering a practical route to sustain cognitive and behavioral engagement for older adults with mobility or access limitations [58]. Recent evidence further underscores the prognostic importance of apathy as a distinct neuropsychiatric syndrome after stroke. In a large prospective study of 422 ischemic stroke patients, both pre-stroke and early post-stroke apathy independently predicted dementia at 3-month follow-up, even after adjusting for age, stroke severity, and delirium, whereas depressive symptoms were not associated with cognitive decline [59]. These findings, although derived from a limited number of trials within this review, are supported by broader observational evidence indicating that apathy may represent an early behavioral marker of post-stroke dementia, emphasizing the need for timely recognition and targeted motivational interventions in secondary prevention.

Finally, implementation matters. Cognitive–communication frameworks from clinical practice stress that therapy works best when education is continuous, caregiver-inclusive, and mapped to instrumental activities of daily living (e.g., medication scheduling, appointments, budgeting, meal preparation) [11]. This aligns with the included RCT pool, where family/peer involvement and structured feedback consistently bolstered adherence. Beyond information transfer, education serves as a therapeutic process that supports both cognitive and emotional recovery; when delivered as structured, goal-oriented learning tailored to individual capabilities, it reinforces neural relearning, fosters autonomy, and links cognitive rehabilitation to day-to-day functioning. Embedding literacy-sensitive, relationship-centered education within nurse-led or community programs therefore provides the foundation for sustainable self-management and resilience in the long-term stroke journey.

#### 4.2.5. Rehabilitation and Physical Activity Approaches

Rehabilitation and structured physical activity remain essential components of secondary stroke prevention, advancing both vascular risk reduction and functional recovery. Contemporary evidence shows that aerobic and strength training are associated with improvements in cardiorespiratory fitness, mobility, and, in some studies, executive function while reducing fatigue and depressive symptoms [15,60,61,62,63]. However, participation among older adults remains suboptimal, constrained by fear of recurrent stroke, comorbidities, and access barriers. In the included RCTs, multicomponent rehabilitation models integrating physical training with behavioral coaching, feedback and education achieved greater adherence and quality-of-life gains than exercise-only programs, underscoring the need for motivational and educational scaffolding alongside physical reconditioning, especially for older adults managing complex health needs [20,29,34,54].

Evidence from community and home-based exercise trials indicates that guideline-level, moderate-intensity aerobic activity of about 150 min per week can safely improve cardiorespiratory fitness and often lowers systolic blood pressure when appropriately monitored [63]. Importantly, nurse-supervised and telerehabilitation models have demonstrated comparable outcomes to center-based physiotherapy, offering scalable options for rural or mobility-limited populations [64,65]. These findings align with current AHA/ASA recommendations that advocate structured, goal-oriented physical activity as part of comprehensive, interdisciplinary secondary prevention plans [6].

Beyond physical outcomes, exercise contributes to neuroplastic recovery and psychosocial well-being. Combined aerobic and cognitive dual-task training can improve executive control and everyday problem-solving, while group-based exercise fosters social engagement and motivation, factors strongly linked to sustained participation [15,63]. Embedding such programs within community or primary care networks, with individualized progression and caregiver involvement, maximizes feasibility and sustainability for older survivors. Overall, integrating rehabilitation and physical activity into the continuum of secondary prevention positions exercise as a lifelong strategy supporting autonomy, confidence, and resilience after stroke [66].

### 4.3. Clinical and Policy Implications

#### 4.3.1. Clinical Implications

The findings of this review suggest that effective secondary stroke prevention in older adults benefits from integrated, person-centered care models that extend beyond hospital settings. While the AHA/ASA 2021 Guideline highlights persistent concerns about suboptimal adherence to antithrombotic, antihypertensive, and lipid-lowering therapy among stroke survivors [6], multiple interventions synthesized in this review, lifestyle and behavioral programs such as HEALS [33] and self-management approaches summarized by Cadel et al. [67], demonstrated that individualized and continuous follow-up, education, structured home visits, and goal-setting can meaningfully strengthen long-term prevention. The PREVENT trial further reinforced that structured exercise combined with education programs sustain long-term improvements in physical function and secondary prevention behaviors [34]. Beyond the included studies, nurse-led post-stroke care models such as SOS-Care [68] illustrate how case management and coordinated follow-up can bridge the post-discharge gap for older adults. Together, these models align with the WHO Rehabilitation 2030: A Call for Action [11], emphasizing that rehabilitation and secondary prevention should be embedded in primary care through multidisciplinary collaboration.

Clinical implementation should also integrate behavioral and educational strategies into routine follow-up and rehabilitation pathways, addressing the practical barriers to medication persistence, physical activity, and diet that are common in older stroke survivors. Evidence from behavioral interventions such as HEALS [33] and MaMoRS [26] supports the use of goal-setting, motivational interviewing, and caregiver involvement as effective tools for improving long-term compliance and vascular risk control. Culturally tailored self-management models designed for minority populations [31] and pragmatic home-care interventions [30] demonstrate how context-adapted education and support can enhance engagement in resource-limited settings. Collectively, these strategies emphasize the need for structured, behaviorally informed education embedded within ongoing rehabilitation and primary care, rather than isolated one-time counseling sessions [69]. This approach is especially relevant for nursing homes and rural areas, where limited staffing and fragmented communication continue to hinder the continuity of preventive care and the implementation of long-term adherence strategies.

Another important implication is the need to recognize and manage cognitive and functional problems that frequently affect older stroke survivors. Difficulties with memory, planning, or motivation, such as executive dysfunction, mild cognitive impairment, or post-stroke apathy, can interfere with how consistently patients follow medication and lifestyle advice. Routine cognitive screening with brief, validated tools can help detect such problems early and is consistent with the European Stroke Organization’s 2030 Action Plan [7]. Beyond screening, exercise-based rehabilitation programs should also deliberately include aerobic and strength components adapted to frail adults: in the trial by Deijle et al. [20], year-long aerobic and resistance training improved cognitive performance after TIA or minor stroke, while the PREVENT program [34] showed that combining exercise with education supported lasting functional recovery. Targeted cognitive interventions also show preliminary promise, although the evidence base remains limited and largely derived from small trials: decision neuroscience informed training enhanced cognitive recovery [24], while inpatient strategy training reduced post-stroke apathy, a barrier to long-term engagement [32]. Altogether, multidisciplinary rehabilitation pathways that integrate medical risk-factor management with structured cognitive, psychological, and occupational components are likely to lower recurrence risk while maintaining independence and quality of life among older adults living with stroke.

Finally, digital health technologies are becoming important complements to routine secondary prevention. Evidence from recent trials, including the mobile health SINEMA program in China [28], the MaMoRS application in India [26], and telemonitoring initiatives such as TASMIN5S IRL [21], shows that digital tools can improve medication tracking, blood pressure control, and follow-up engagement. Their long-term value, however, depends on how easily older adults can use them and on whether patients and caregivers receive adequate training. Embedding these tools within structured post-stroke follow-up systems, as demonstrated by Doogue et al. [21] and Yan et al. [28], could help reduce age-related disparities and translate evidence-based guidelines into everyday practice.

#### 4.3.2. Policy Implications

Translating these clinical insights into sustainable policy requires that secondary stroke prevention be viewed not only as a medical responsibility but as a coordinated public health priority. The European Stroke Organization and the Stroke Alliance for Europe have emphasized in their Stroke Action Plan for Europe 2018–2030 that integrated prevention and rehabilitation pathways should be embedded within national health strategies and supported by long-term funding mechanisms [5,7]. This review highlights the need for policies that strengthen the interface between hospital and community services, particularly for older adults who face social isolation, frailty, or limited access to specialized care. National stroke frameworks should prioritize workforce training in behavioral and cognitive rehabilitation, invest in digital infrastructure for telerehabilitation and remote monitoring [42], and ensure reimbursement for multidisciplinary follow-up visits. Aligning these efforts with the WHO Rehabilitation 2030: A Call for Action [11] and the AHA/ASA 2021 Guidelines [6] would help standardize care across regions while promoting equitable access to prevention programs. Finally, the SAFE Stroke Action Plan for Europe 2018–2030 underscores that policy evaluation and patient-reported outcomes are essential to ensure accountability and long-term impact [70]. Health policy frameworks should therefore prioritize continuity of care rather than episodic contact, creating systems where every stroke survivor, regardless of age or geography, remains connected to long-term, person-centered support.

### 4.4. Strengths and Limitations of the Study

This systematic review has several strengths. It followed a transparent methodology aligned with PRISMA 2020 guidelines [17] and focused exclusively on randomized controlled trials (RCTs) addressing secondary stroke prevention in older adults, a population often underrepresented in research. The systematic search was conducted across two major databases (PubMed and Scopus), ensuring broad coverage of relevant literature. Independent screening of all records by four reviewers minimized selection bias and increased methodological transparency. The included randomized trials represented a wide geographical diversity, spanning North America, Europe, Asia, and Africa, which enhances the external validity and cross-cultural applicability of the findings. Moreover, the synthesis covered diverse intervention types, including behavioral, cognitive, exercise-based, and digital, offering a comprehensive perspective on multidisciplinary approaches to post-stroke care.

However, some limitations should also be acknowledged. Study quality, appraised with RoB 2 [18], was generally acceptable: no trial was rated at high risk of bias, although many were classified as some concerns owing to limited reporting of allocation concealment or blinding. These reporting gaps, common in behavioral/rehabilitation research, may introduce some uncertainty, although the overall direction of effects appeared consistent across studies. We therefore interpret pooled patterns cautiously, emphasizing consistency across settings rather than isolated point estimates. The heterogeneity of study designs, outcome measures, and follow-up durations precluded a quantitative meta-analysis and limited cross-study comparability. Most trials involved short-term follow-up periods, and few reported cost-effectiveness or implementation fidelity outcomes. Restricting the search to English-language publications and excluding gray literature might have introduced publication bias. An additional limitation is that the electronic search was restricted to only two major databases (PubMed and Scopus). Although these platforms provide extensive biomedical and multidisciplinary coverage and have been shown to retrieve a high proportion of relevant RCTs, this approach may still limit the comprehensiveness of the review for a global audience. Studies indexed exclusively in other databases such as Embase, Web of Science, CINAHL, or regional repositories may not have been captured. Therefore, the findings should be interpreted with this methodological constraint in mind. Furthermore, most included RCTs did not report outcomes stratified by sex, preventing an analysis of gender-related differences in risk factors, intervention effects, or stroke severity. This gap limits the ability to assess well-established sex-specific patterns in secondary stroke prevention. Despite these limitations, the review contributes valuable insight into the complex, multifaceted nature of secondary stroke prevention in older adults and provides a foundation for future multidisciplinary and educational research aimed at improving long-term outcomes in this population.

### 4.5. Future Research Directions

Future research should move beyond evaluating isolated interventions and focus on developing comprehensive, multidisciplinary models that reflect the complex realities of older stroke survivors. A key priority is to redefine education not as a passive transfer of information but as an active, therapeutic process that promotes behavioral change, cognitive engagement, and emotional adaptation. Education should be studied as a structured, goal-oriented intervention integrated into rehabilitation, one that empowers patients to understand, internalize, and actively apply prevention strategies in daily life. This approach demands collaboration among educators, nurses, therapists, psychologists, and physicians to ensure that teaching methods address both cognitive limitations and motivational needs.

Future studies should also explore how educational therapy can be effectively combined with exercise, digital monitoring, and psychosocial support to reinforce adherence and long-term recovery. Pragmatic and hybrid study designs, implemented in diverse real-world community and long-term care settings, are needed to evaluate sustainability, accessibility, and equity of these models. Moreover, involving caregivers and patients as active partners in research could yield deeper insight into what makes educational interventions meaningful and lasting. In aging populations, such educational frameworks could play a central role in mitigating stroke-related disability and promoting active, autonomous aging.

In addition, future research should systematically incorporate sex-stratified analyses. Most existing RCTs do not report outcomes separately for men and women, limiting the ability to understand known sex-specific differences in post-stroke risk profiles, treatment responses, and recovery patterns. Addressing this gap is essential for developing more precise and equitable secondary prevention strategies for older adults.

Future research should also focus specifically on very old stroke survivors (≥80–85 years), who represent a rapidly growing population segment yet remain markedly underrepresented in randomized trials. Existing evidence suggests that the clinical profile, comorbidity burden, functional reserve, and risk-factor patterns differ substantially in this age group, indicating that secondary prevention strategies may require age-tailored adaptation. Dedicated RCTs and subgroup analyses are needed to clarify which interventions are most effective, feasible, and safe for the oldest adults.

Ultimately, future research could help position education as a cornerstone of secondary stroke prevention, an evidence-based therapeutic act that bridges medicine, rehabilitation, and lifelong learning. Establishing this framework could form the basis for a new generation of stroke rehabilitation programs, where learning itself becomes part of healing.

## 5. Conclusions

In conclusion, this systematic review found that effective secondary stroke prevention in older adults relies on integrated, person-centered approaches that extend beyond pharmacological management. The evidence synthesized from recent randomized controlled trials demonstrates that interventions combining education, behavioral change, physical activity, cognitive rehabilitation, and digital monitoring can meaningfully reduce recurrence risk and improve long-term functional recovery. By focusing on older adults, a population often overlooked in clinical research, this review underscores the importance of adapting prevention strategies to the unique cognitive, motivational, and social realities of aging. Beyond clinical management, the findings point to the therapeutic potential of structured educational programs that target cognition, motivation, and self-efficacy as integral components of recovery.

Looking ahead, advancing stroke prevention will depend on how effectively healthcare systems integrate these educational and multidisciplinary approaches into everyday practice. Prioritizing continuous, personalized, and accessible models of care can help bridge the gap between acute treatment and long-term support, particularly for aging populations. As the global burden of stroke continues to rise, empowering older adults through knowledge, motivation, and coordinated care may ultimately prove to be the most sustainable path toward prevention and long-term well-being.

Recent advances in neuroscience further suggest that the aging brain retains substantial capacity for neuroplasticity and reorganization. Complex cognitive training has been shown to enhance cerebral blood flow, white matter integrity, and functional connectivity in healthy seniors [71]. These findings indicate that recovery potential after stroke may depend not only on pharmacological and behavioral management but also on how rehabilitation intensity and personalization engage neural mechanisms of plasticity. Future research should explore how age-related and stroke-related brain changes interact, and how tailored, multidisciplinary interventions can best harness this capacity for restoration and resilience in older adults. Integrating this neurobiological perspective into rehabilitation and educational interventions could guide the development of next-generation, evidence-based models of secondary prevention for aging populations.

## Figures and Tables

**Table 1:** PICO framework used to define eligibility criteria and guide study selection.

Element	Description
P—Population	Older adults aged 60 years and above who had experienced an ischemic or hemorrhagic stroke or transient ischemic attack (TIA). Studies including mixed-age populations were eligible if results for older participants could be clearly identified or if the mean sample age was ≥60 years.
I—Intervention	Any intervention aimed at secondary prevention of stroke recurrence, including: Pharmacological management (antiplatelet, antihypertensive, lipid-lowering therapy, anticoagulant therapy, or fixed-dose combinations). Behavioral or educational interventions (patient education, self-management, motivational interviewing, lifestyle modification). Rehabilitation and physical activity interventions. Technology-assisted or mHealth interventions (telemonitoring, mobile apps). Nurse-led, physician-led, or multidisciplinary care models.
C—Comparator	Usual care, standard clinical management, placebo, or control conditions not involving structured secondary prevention programs.
O—Outcomes	Primary outcomes: Medication adherence Stroke recurrence Vascular risk-factor control (blood pressure and lipid management) Secondary outcomes: Lifestyle modification (physical activity, diet, smoking cessation) Functional recovery and quality of life Cognitive and psychological outcomes (e.g., self-efficacy, depression) Implementation-related factors (barriers and facilitators to adherence and engagement)

**Table 3:** Summary of Included Randomized Controlled Trials.

Author, (Year)	Country	Population	Intervention/Focus	Main Outcome	Key Findings
Appalasamy et al. (2020) [25]	Asia (Malaysia)	54 post-stroke outpatients	Video narratives designed to improve patients’ understanding and medication adherence	Medication Understanding and Use Self-Efficacy (MUSE); Systolic Blood Pressure	The video-based intervention was feasible and well accepted. Participants showed a significant increase in medication understanding and self-efficacy (*p* = 0.001) and a significant reduction in systolic blood pressure after three months (*p* = 0.04).
Babu (2024) [26]	Asia (India)	209 stroke survivors	Smartphone application providing medication reminders, health education, and lifestyle guidance	Medication adherence; Risk-factor control	The intervention group demonstrated higher medication adherence (*p* < 0.001), improvements in diet and physical activity, lower fasting blood sugar (*p* = 0.005), and higher HDL cholesterol (*p* = 0.024) after six months.
Damush (2011) [29]	USA	63 recently hospitalized veterans with acute ischemic stroke	12-week stroke self-management program vs. attention-control	Self-efficacy; aerobic exercise; stroke-specific quality of life	Improved self-efficacy, weekly aerobic minutes, and selected SS-QoL domains vs. control; feasible pilot within VA system.
Deijle (2024) [20]	Europe (Netherlands)	119 patients with TIA or minor ischemic stroke	One-year combined aerobic and strength training program focused on cognitive recovery	Executive functioning	The program led to significant and sustained improvements in executive functioning (β = 0.13, *p* = 0.03), while no notable effects were found for memory or attention-psychomotor speed.
Doogue (2023) [21]	Europe (Ireland)	15 post-stroke/TIA adults with uncontrolled SBP (>130 mmHg)	Integrated home BP self-monitoring + pre-agreed GP titration (BP: Together) vs. usual care	Systolic and diastolic blood pressure	Feasible and acceptable; greater SBP (−23 mmHg) and DBP (−10 mmHg) reductions vs. control at 3 months; no adverse events reported.
Feldman (2020) [30]	USA	495 Black/Hispanic post-stroke/TIA patients with uncontrolled SBP	Usual home care vs. nurse practitioner transitional care ± health coach	Systolic blood pressure at 3 and 12 months	All groups had ~10 mmHg SBP reductions; NP or NP + HC added no benefit over usual home care; pragmatic real-world feasibility in disadvantaged populations.
Geary (2019) [22]	Europe (Sweden)	Primary-care physicians; outcomes in 12,766 stroke/TIA patients	Audit-and-feedback to physicians on secondary prevention prescribing	Medication dispensation/adherence at 18 months	No improvement in medication dispensation vs. control; one-off feedback and high baseline use likely limited effect.
Hornnes (2011) [23]	Europe (Denmark)	349 recent stroke or TIA patients	Nurse-led home visits including blood-pressure monitoring and personalized lifestyle counseling	Systolic blood pressure at one year	The intervention did not significantly reduce systolic blood pressure at one year but increased antihypertensive prescriptions and patient compliance with general practitioner follow-ups.
MacKay-Lyons (2022) [34]	North America (Canada)	84 adults (<3 months post non-disabling stroke or TIA)	12-week PREVENT program combining supervised aerobic and strength exercise with weekly education sessions on risk-factor management	Blood pressure; Lipid profile; Fitness	The program did not significantly reduce systolic blood pressure compared with usual care. However, short-term improvements were observed in diastolic pressure and LDL cholesterol. Adherence was high, and the intervention was feasible across urban and rural sites.
Perera (2025) [35]	North America (Canada)	101 recent ischemic stroke/high-risk TIA with ICAD	Low-dose rivaroxaban (2.5 mg bid) + aspirin vs. aspirin alone	Ischemic stroke + covert infarcts (MRI); safety	Safe regimen; non-significant trend to fewer events vs. aspirin; multicenter feasibility demonstrated, supporting a larger phase III trial.
Sajatovic (2018) [31]	USA	38 African American men with prior stroke/TIA	TEAM self-management (1 individual + 4 group sessions) vs. treatment as usual	Systolic BP; HbA1c; HDL	Lower SBP at 24 weeks and improvements in HbA1c and HDL in TEAM; qualitative data indicated better risk awareness and peer support.
Sarfo (2023) [36]	Africa (Ghana)	Approximately 200 patients with recent ischemic stroke	Polypill combining aspirin, statin, and antihypertensive agents for secondary prevention	Carotid intima-media thickness; Vascular risk-factor control	The polypill showed no superiority over usual care in carotid intima-media thickness or secondary outcomes. Blood pressure and LDL levels were similar between groups, while adverse events were more frequent in the polypill group.
Skidmore (2015) [32]	USA	30 inpatients with acute stroke and cognitive impairments	Strategy training (Goal-Plan-Do-Check) vs. reflective listening (attention control)	Apathy symptoms over 6 months	Strategy training yielded lower apathy scores at 3 and 6 months vs. control; suggests benefit as adjunct during inpatient rehabilitation.
Studer (2021) [24]	Europe (Germany/UK)	83 post-acute stroke with visuospatial WM deficits	Precommitment + daily 30 min gamified training (“Wizard”) vs. training-only vs. standard therapy	Training adherence; working memory	Precommitment increased adherence and total training dose; Wizard users improved visuospatial and verbal working memory.
Towfighi (2020) [33]	USA	100 socioeconomically disadvantaged stroke/TIA survivors	HEALS: 6-week OT-led group lifestyle program vs. usual care	BMI; diet; physical activity at 6 months	Feasible and acceptable, but no significant changes in BMI, diet, or activity; formative feedback supports longer, standardized, family-inclusive delivery.
Yamagami (2024) [27]	Asia (Japan)	631 with carotid stenosis undergoing CAS	Add-on cilostazol vs. standard antiplatelet therapy	In-stent restenosis within 2 years	Reduced ISR beyond 30 days with cilostazol; primary endpoint trend non-significant; acceptable safety; underpowered, larger trials warranted.
Yan (2021) [28]	Asia (China—rural)	1299 stroke survivors	Primary-care mHealth program combining physician training, app-based follow-up, and daily voice messages promoting adherence and activity	Systolic blood pressure; Stroke recurrence	The intervention achieved significant reductions in systolic blood pressure (*p* = 0.005), improvements in multiple secondary outcomes including quality of life and medication adherence, and lower rates of stroke recurrence, hospitalization, and mortality.

## Data Availability

No new data were created.

## Supplementary Materials

The following supporting information can be downloaded at: https://doi.org/10.5281/zenodo.17565301 (accessed on 7 December 2025). Table S2: Risk-of-bias assessment of included randomized controlled trials using the RoB 2 tool; Table S3: Detailed signaling questions and justifications for risk-of-bias judgments; Table S4: PRISMA 2020 Checklist for the Systematic Review.
